# Copy Number Alterations in Hepatoblastoma: Literature Review and a Brazilian Cohort Analysis Highlight New Biological Pathways

**DOI:** 10.3389/fonc.2021.741526

**Published:** 2021-12-08

**Authors:** Juliana Sobral Barros, Talita Ferreira Marques Aguiar, Silvia Souza Costa, Maria Prates Rivas, Monica Cypriano, Silvia Regina Caminada Toledo, Estela Maria Novak, Vicente Odone, Lilian Maria Cristofani, Dirce Maria Carraro, Isabela Werneck da Cunha, Cecília Maria Lima Costa, Angela M. Vianna-Morgante, Carla Rosenberg, Ana Cristina Victorino Krepischi

**Affiliations:** ^1^Human Genome and Stem Cell Research Center, Department of Genetics and Evolutionary Biology, Institute of Biosciences, University of São Paulo, São Paulo, Brazil; ^2^Department of Urology, New York University (NYU) Grossman School of Medicine, New York, NY, United States; ^3^Department of Pediatrics, Institute of Pediatric Oncology, Support Group for Children and Adolescents with Cancer (IOP-GRAACC), Federal University of São Paulo, São Paulo, Brazil; ^4^Department of Pediatrics, Institute of Childhood Cancer Treatment (ITACI), Faculty of Medicine, University of São Paulo, São Paulo, Brazil; ^5^International Research Center, AC Camargo Cancer Center (ACCCC), São Paulo, Brazil

**Keywords:** copy number alteration, hepatoblastoma, pediatric cancer, cytogenomics, array-CGH

## Abstract

Hepatoblastoma (HB) is a rare embryonal tumor, although it is the most common pediatric liver cancer. The aim of this study was to provide an accurate cytogenomic profile of this type of cancer, for which information in cancer databases is lacking. We performed an extensive literature review of cytogenetic studies on HBs disclosing that the most frequent copy number alterations (CNAs) are gains of 1q, 2/2q, 8/8q, and 20; and losses at 1p and 4q. Furthermore, the CNA profile of a Brazilian cohort of 26 HBs was obtained by array-CGH; the most recurrent CNAs were the same as shown in the literature review. Importantly, HBs from female patients, high-risk stratification tumors, tumors who developed in older patients (> 3 years at diagnosis) or from patients with metastasis and/or deceased carried a higher diversity of chromosomal alterations, specifically chromosomal losses at 1p, 4, 11q and 18q. In addition, we distinguished three major CNA profiles: no detectable CNA, few CNAs and tumors with complex genomes. Tumors with simpler genomes exhibited a significant association with the epithelial fetal subtype of HBs; in contrast, the complex genome group included three cases with epithelial embryonal histology, as well as the only HB with HCC features. A significant association of complex HB genomes was observed with older patients who developed high-risk tumors, metastasis, and deceased. Moreover, two patients with HBs exhibiting complex genomes were born with congenital anomalies. Together, these findings suggest that a high load of CNAs, mainly chromosomal losses, particularly losses at 1p and 18, increases the tendency to HB aggressiveness. Additionally, we identified six hot-spot chromosome regions most frequently affected in the entire group: 1q31.3q42.3, 2q23.3q37.3, and 20p13p11.1 gains, besides a 5,3 Mb amplification at 2q24.2q24.3, and losses at 1p36.33p35.1, 4p14 and 4q21.22q25. An *in-silico* analysis using the genes mapped to these six regions revealed several enriched biological pathways such as ERK Signaling, MicroRNAs in Cancer, and the PI3K-Akt Signaling, in addition to the WNT Signaling pathway; further investigation is required to evaluate if disturbances of these pathways can contribute to HB tumorigenesis. The analyzed gene set was found to be associated with neoplasms, abnormalities of metabolism/homeostasis and liver morphology, as well as abnormal embryonic development and cytokine secretion. In conclusion, we have provided a comprehensive characterization of the spectrum of chromosomal alterations reported in HBs and identified specific genomic regions recurrently altered in a Brazilian HB group, pointing to new biological pathways, and relevant clinical associations.

## Introduction

Hepatoblastoma (HB) is a very rare childhood cancer, accounting for approximately 1% of all pediatric tumors, with an annual incidence of 2.16 per million cases (1); however, it is the most common primary liver tumor in children, generally diagnosed in the first 3 years of life (2). Most HBs are sporadic, although it can occur in association with genetic syndromes, such as Familial Adenomatous Polyposis, Beckwith–Wiedemann syndrome (BWS), Trisomy 18 (Edwards Syndrome), and other inherited syndromes (3), in addition to a well-documented association with congenital abnormalities (4). This embryonal tumor is probably originated from undifferentiated hepatocyte precursor cells, since its histology recapitulates liver embryonic development, showing a combination of histological patterns from different stages of cell differentiation (5).

Alterations involving gene activators of the WNT signaling pathway are frequent, mainly *CTNNB1* mutations, and rare *AXIN1*, *AXIN2*, and *APC* alterations; *TERT* promoter (2) and *NFE2L2* mutations (6) were also reported. Another molecular mechanism known to be involved in HB development are imprinting defects at the BWS critical region (11p15), resulting in *IGF2* overexpression and *H19* downregulation (7); loss of heterozygosity (LOH) at 11p15 was also observed in some sporadic HBs, as well as in other embryonal tumors (8).

Cytogenetically, HB is characterized by copy number alterations involving gains of 1q, 2/2q, 8/8q and 20, as well as 4q and chromosome 18 losses (9, 10). A recurring translocation of chromosomes 1 and 4 was reported by Schneider et al. (11), resulting in a derivative chromosome [der(4)t(1,4) (q12; q34)]. In spite of the acknowledgment about these genomic alterations, their contribution to the molecular mechanisms involved in tumorigenesis and progression of HBs are poorly explored.

In this study, an extensive literature review of 45 published articles on HB cytogenetics/cytogenomics was performed, aiming to evaluate the profile of copy number alterations (CNA) in this type of cancer and their frequencies. Additionally, we investigated the CNA profile of a Brazilian cohort of 26 HBs, in an attempt to further define genomic regions of potential relevance in this tumor type.

## Materials and Methods

### Patients

This study was carried out through the collaboration of three clinical research groups of childhood cancer in São Paulo, Brazil: A.C Camargo Cancer Center (ACCCC), the Institute of Pediatric Oncology (Support Group for Children and Adolescents with Cancer (IOP-GRAACC), and the Institute of Childhood Cancer Treatment (ITACI). The study was approved by the Research Ethics Committees of the involved institutions, and informed consents were obtained from the patients’ parents or legal guardians. The cohort consisted of 26 HB tumor samples, including the reanalysis at higher resolution of samples previously reported (12). **Table 1** presents the clinical features of each patient, and their respective tumors.

**Table 1:** Clinical information of 26 Brazilian patients with hepatoblastoma.

ID	Age at diagnosis	Sex	Histology	AFP ng/mL	PRETEXT	Risk stratification**	Metastasis	Relapse	Deceased
HB15T*	1.5 years	F	Epithelial Embryonal	5668000	4	Intermediate	No	No	Yes
HB16T*	9 months	M	Epithelial Fetal	824	4	Intermediate	No	No	No
HB17T*	3 years	F	Epithelial Fetal	>400000	1	Low	No	No	No
HB18T*	9 months	M	Epithelial and Mesenchymal mixed	>200000	3	Low	No	No	No
HB28T*	17 years	M	Epithelial and Mesenchymal mixed	NA	4	High	No	Yes	Yes
HB30T*	4.5 years	M	HB with HCC features	>1000000	2	High	Pulmonary	Yes	Yes
HB31T*	2.5 years	M	Epithelial Fetal	742000	3	Low	No	No	No
HB32T*	3 years	F	Epithelial and Mesenchymal mixed	9328000	4	High	Pulmonary	No	No
HB33T*	1 month	F	Epithelial Embryonal and Fetal	28312000	2	Intermediate	No	No	No
HB34T	1.5 years	F	Epithelial Fetal	416430	3	Intermediate	No	No	No
HB35T	2 years	M	Epithelial Fetal	54800	3	Intermediate	No	No	No
HB36T	2.5 years	M	Epithelial Fetal	76348	3	Low	No	No	No
HB38T	12 years	F	Epithelial Fetal	643,4	4	High	No	Yes	No
HB39T	7 years	M	Epithelial and Mesenchymal mixed	>300.000	2	High	No	No	Yes
HB40T	1.8 years	M	Epithelial Embryonal and Fetal	1842,6	1	Low	No	No	No
HB41T	1.7 years	M	Epithelial Fetal	201733	3	High	Pulmonary	No	No
HB42T	3.7 years	M	Epithelial Fetal	1267	1	Low	No	No	No
HB43T	1.7 years	M	Epithelial Embryonal	183476	4	Intermediate	No	No	No
HB44T	5 months	M	Epithelial and Mesenchymal mixed	>300000	2	Intermediate	No	No	No
HB45T	5 months	F	Epithelial Fetal	445611	2	Low	No	No	Yes
HB46T	2.3 years	M	Epithelial and Mesenchymal mixed	>200000	4	High	Pulmonary	No	No
HB66T	10 months	M	Epithelial Embryonal	409596	2	High	Pulmonary	Yes	Yes
HB70T	6 years	F	Epithelial Fetal	46809	2	High	Pulmonary	Yes	Yes
HB72T	5 months	M	Epithelial Fetal	2565530	4	Intermediate	No	No	No
HB79T	1.5 years	M	Epithelial Fetal	>50000	4	High	No	No	No
HB81T	1.7 years	M	Epithelial and Mesenchymal mixed	>100000	4	High	Pulmonary	No	No

F, Female; M, Male; NA, Not Available; AFP, Alpha-fetoprotein; PRETEXT, Pretreatment extent of disease.

*Previously published by Rodrigues et al. (12).

**Risk stratification according to CHIC: The Children’s Hepatic tumors International Collaboration - Czauderna et al. (13); Meyers et al. (14).

DNA was extracted from fresh frozen tumor tissue (specimen of surgery) samples, following standard technical procedures, using QIASymphony DNA Mini kit (QIAGEN).

### Chromosomal Microarray Analysis (CMA)

CMA was performed using a 180K array-CGH platform (Agilent Technologies), following established protocols. Generated data were analyzed for CNAs using the Nexus Copy Number 9.0 software (BioDiscovery), with the FASST2 Segmentation algorithm, threshold log2 Cy3/Cy5 ratio of |0.1| for gains and losses (allowing the detection of mosaicism), and |1.2| for amplifications and homozygous losses. Common germline CNVs were disregarded based on the comparison with the Database of Genomic Variants (http://dgv.tcag.ca/dgv/app/home). The human genome annotation was based on the GRCh37/hg19 from the Genome Browser at the University of California Santa Cruz - UCSC (https://genome.ucsc.edu/).

### *In Silico* Analysis of Genes and Biological Pathways

Gene lists were evaluated using the VarElect tool (https://ve.genecards.org/), with the keywords “cancer” or “hepatoblastoma”. A gene set with the highest scores (top 10% cut-off; score > 6) was selected for an *in-silico* analysis using the online tool GeneAnalytics (http://geneanalytics.genecards.org/).

## Results

### Copy Number Alterations in the Brazilian HB Cohort

**Figure 1:**
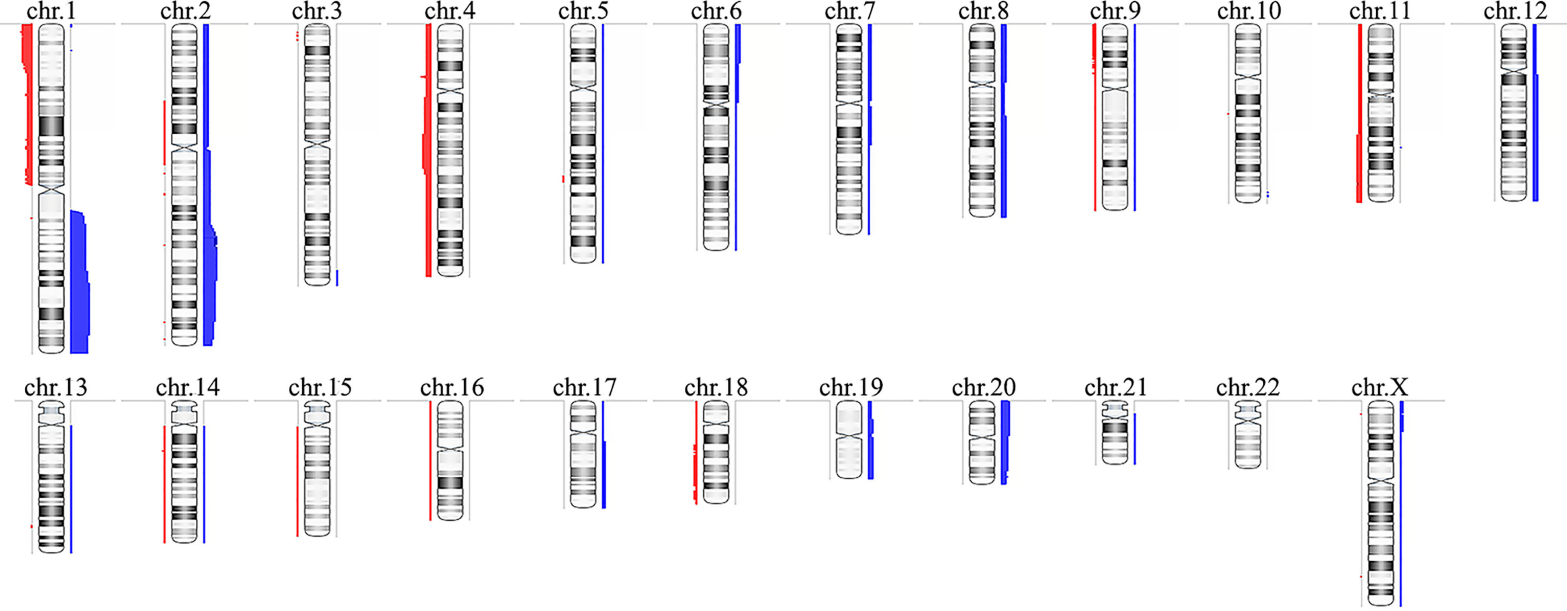
Copy number alterations profile of the Brazilian hepatoblastoma cohort investigated by chromosomal microarray analysis using aCGH. Ideograms of chromosomes are depicted with copy number gains represented in blue bars at right, and losses in red at left; the width of the bars indicates the frequency of the alteration in the entire group. Chromosome Y was not evaluated. Image generated using the software Nexus Copy Number 9.0 (BioDiscovery).

CMA revealed alterations in 16 of the 26 HB samples, identifying a total of 121 CNAs in the cohort (**Supplementary Table 1**), corresponding to ~4.6 CNAs per tumor, with a median size of 44.2 Mb (mean 56.7 Mb). Fifty-nine copy number gains were identified, ranging from 467 kb to 243 Mb (entire chromosome), with a median size of 77.1 Mb (mean 81.2 Mb); sixty-two losses were identified, ranging from 123.7 kb to 191 Mb (entire chromosome), with a median size of 2 Mb (mean 33.4 Mb). A schematic view of the CNA profile of the Brazilian HB cohort is shown in **Figure 1**. Among the 16 tumors carrying CNAs, the most frequent alterations were gains affecting 1q (61%), 2/2q (44%) and 20/20p (28%), while losses were predominant at 1p/1pter (44%) and 4/4q (28%).

**Figure 2:**
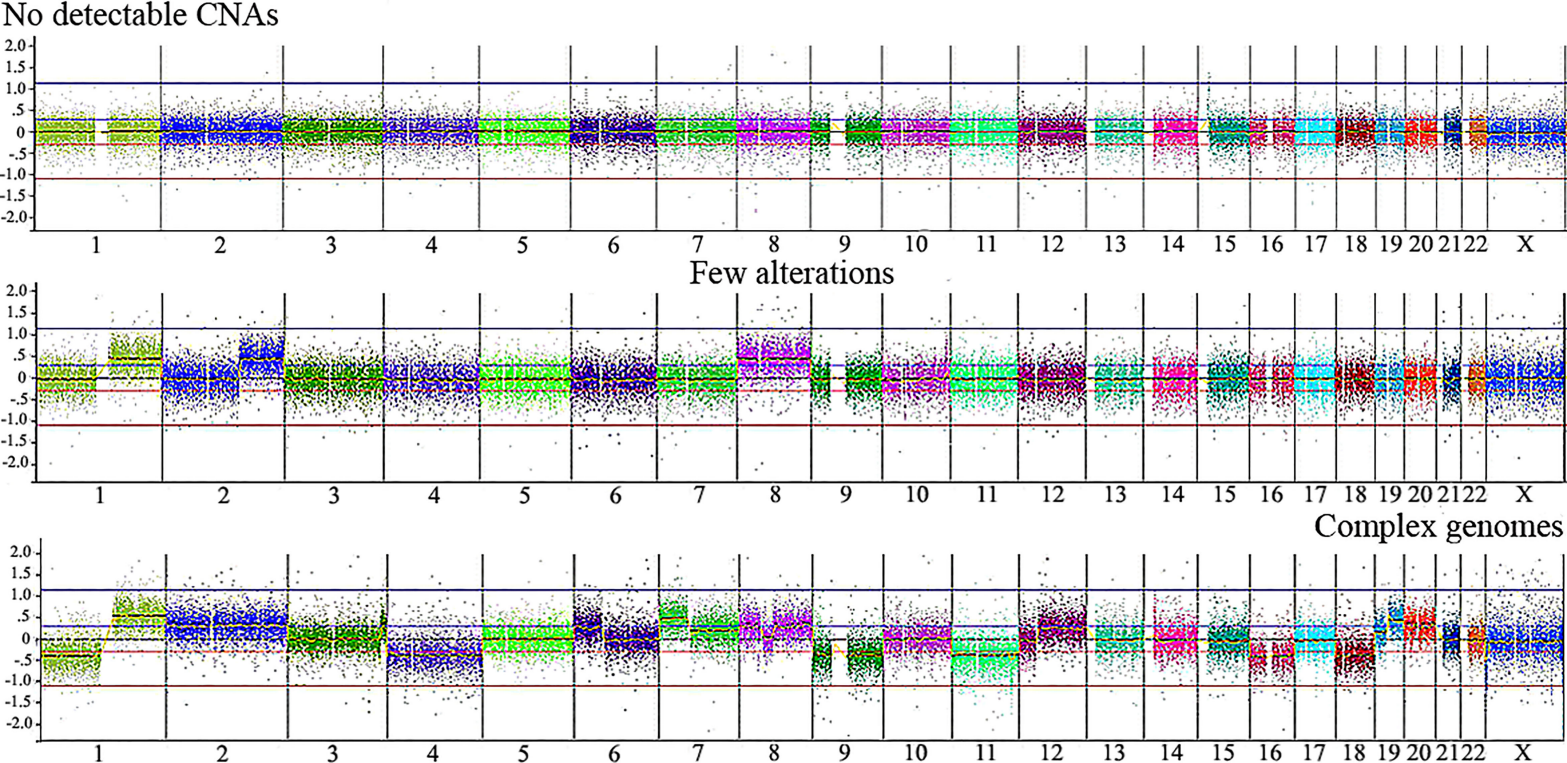
Illustrative CNA profiles representing the three categories of tumors identified by aCGH in the Brazilian HB cohort. No detectable CNA (HB42T), few alterations (HB43T) and complex genomes (HB70T). Microarray probes are plotted according to their genomic coordinates, each color representing a chromosome, from 1 to 22, X, from the short arm to the long arm. The Y axis represents the log_2_ scale of the copy number ratio tumor/control (values close to 0 indicate regions with similar copy number between tumor and control samples; positive values represent gains; negative values represent losses).

**Table 2** presents CNA types and absolute number detected in each tumor, as well as summarized clinical details; the immunohistochemistry information regarding *CTNNB1* activation was retrieved from Aguiar et al. (15). We found significant associations with patient’s age at diagnosis < 3 and the presence of *CTNNB1* activation (p value < 0.007), and patient’s age at diagnosis > 3 years and death from the disease (p value <0.028). Three categories of cytogenomic profile were defined based on the empirical observation of our own CNA data (**Figure 2** and **Supplementary Table 2**). Considering only the 16 tumors with detectable CNAs, the mean CNA number was 7.6. Tumors with < 7.6 CNAs were classified as samples with few alterations (10 tumors: HB16T; HB17T, HB18T, HB36T, HB39T, HB40T, HB41T, HB43T, HB44T, HB79T), and tumors carrying > 7.6 CNAs were classified as complex genomes (six tumors: HB15T, HB30T, HB33T, HB46T, HB66T, HB70T). The third category was composed of HBs in which no CNAs were detected (10 samples: HB28T, HB31T, HB32T, HB34T, HB35T, HB38T, HB42T, HB45T, HB72T, HB81T).

**Table 2:** Description of number and types of CNAs in each tumor sample as well as summarized clinical features.

ID	Gain	Amplification	Loss	Homozygous loss	Total CNAs	CNA profile	Sex	Age at diagnosis	Risk factor	Metastasis	Status	*CTNNB1* activation (immunohistochemistry)*
HB15T	8	–	3	–	11	complex	F	<3 years	Intermediate	No	deceased	Positive
HB16T	1	–	–	–	1	few CNAs	M	<3 years	Intermediate	No	alive	Positive
HB17T	1	–	–	–	1	few CNAs	F	<3 years	Low	No	alive	Negative
HB18T	1	1	1	–	3	few CNAs	M	<3 years	Low	No	alive	Positive
HB28T	1	–	–	–	1	no CNA	M	>3 years	High	No	deceased	Negative
HB30T	5	–	4	–	9	complex	M	>3 years	High	Yes	deceased	Negative
HB31T	1	–	–	–	1	no CNA	M	<3 years	Low	No	alive	Positive
HB32T	–	–	–	–	–	no CNA	F	<3 years	High	Yes	alive	Positive
HB33T	11	–	2	–	13	complex	F	<3 years	Intermediate	No	alive	Positive
HB34T	–	–	–	–	–	no CNA	F	<3 years	Intermediate	No	alive	Negative
HB35T	–	–	–	–	–	no CNA	M	<3 years	Intermediate	No	alive	Positive
HB36T	2	–	3	–	5	few CNAs	M	<3 years	Low	No	alive	Negative
HB38T	–	–	–	–	–	no CNA	F	>3 years	High	No	alive	Negative
HB39T	2	–	2	–	4	few CNAs	M	>3 years	High	No	deceased	Negative
HB40T	–	–	2	–	2	few CNAs	M	<3 years	Low	No	alive	Positive
HB41T	2	–	–	–	2	few CNAs	M	<3 years	High	Yes	alive	Negative
HB42T	–	–	–	–	–	no CNA	M	>3 years	Low	No	alive	Negative
HB43T	5	–	–	–	5	few CNAs	M	<3 years	Intermediate	No	alive	Positive
HB44T	1	–	2	–	3	few CNAs	M	<3 years	Intermediate	No	alive	Negative
HB45T	–	–	–	–	–	no CNA	F	<3 years	Low	No	deceased	Positive
HB46T	1	–	26	–	27	complex	M	<3 years	High	Yes	alive	Positive
HB66T	4	1	5	–	10	complex	M	<3 years	High	Yes	deceased	Positive
HB70T	12	–	11	1	24	complex	F	>3 years	High	Yes	deceased	Negative
HB72T	–	–	1	–	1	no CNA	M	<3 years	Intermediate	No	alive	Negative
HB79T	1	–	–	–	1	few CNAs	M	<3 years	High	No	alive	Positive
HB81T	–	–	–	–	–	no CNA	M	<3 years	High	Yes	alive	Negative

*Data from Aguiar et al. (15).

**Figure 3:**
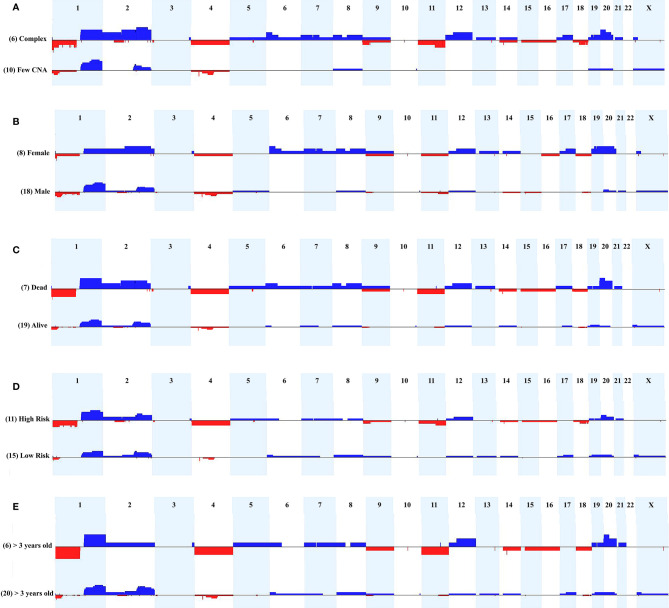
Comparison of CNA profiles of specific clinical features. The X axis displays chromosome profiles from 1 to 22, and X; chromosome Y was not evaluated. Copy number gains are represented in blue bars and losses in red; the width of the bars indicates the frequency of the alteration in the group. **(A)** Tumors with complex genomes versus samples with few CNAs. **(B)** Tumors from females versus tumors from males. **(C)** Tumors from patients who deceased versus tumors from alive patients. **(D)** High-risk versus low-risk tumors. **(E)** Tumors from patients diagnosed > 3 years-old versus samples from patients diagnosed < 3 years-old.

We evaluated the tumor's cytogenomic data looking for relevant differences regarding CNA profiles and clinical parameters such as sex, metastasis, patient’s status (deceased or not), risk stratification, and age at diagnosis. **Figure 3** presents the CNA profiles associated with each of the above-mentioned parameters. We compared the complex genome HB group with the tumors carrying few CNAs, and mainly regions classically reported in HBs were detected in the latter category: gain of chromosomes 1q, 2q, 8 and 20, and 4q loss (**Figure 3A**). Although none specific CNA were enriched in HBs from males or females, the group of HBs from females presented with chromosomal alterations which are distributed along all the genome, including but not restricted to classical HB regions (**Figure 3B**). Three CNAs were enriched in the group of deceased patients (frequency > 35%; p value <0.04): loss of 1p, and gain of 2q proximal and chromosome 20 (**Figure 3C**). Moreover, it can be visualized that patients who deceased and/or had metastasis developed tumors with a higher diversity of CNAs, which were distributed in several different chromosomes. Similarly, high-risk HBs carried a higher frequency and diversity of CNAs, mainly chromosomal losses at 1p (frequency > 35%; p value <0.02), 4, 11q and 18q (**Figure 3D**). HBs from older patients (> 3 years) also exhibited more losses than tumors from younger patients (**Figure 3E**), with 1p deletion presenting a significant difference among these groups (frequency > 35%; p value <0.03). Tumors with *CTNNB1* activation did not present remarkable differences when compared to those with negative *CTNNB1* labelling (**Supplementary Figure 1**).

Additionally, the three CNA profile categories were associated with specific clinical features. The group of 10 tumors with no detectable CNA exhibited enrichment for the epithelial fetal histology (7/10 samples; p value <0.05), and were mostly low/intermediate risk classes (6/10 samples), without relapse, metastasis or fatal outcome. The group of 10 HBs carrying few alterations showed an excess of males (9/10 samples), intermediate/low risk tumors (7/10 samples), and was almost without relapse, metastasis, and death by disease. Opposite to this pattern, the group of six HB samples with complex genomes included three cases of the epithelial embryonal histology, in addition to the only HB with HCC features, and the only congenital HB of the casuistry. Moreover, we observed in the complex genome group a predominance of high-risk samples with significant enrichment for older patients (age at diagnosis > 3 years; p value <0.028), deceased status (p value <0.027) and presence of metastasis (p <0.027).

We also compared the cytogenomic data with the mutational status reported in our previous work based on exome sequencing (15), which presented a partial overlap of HB samples. Tumors with complex genomes also presented a higher number of somatic coding SNV/indel mutations when compared to the average of ~6 somatic mutations/sample observed in the whole group of HBs (HB15T = 17 mutations, HB30T = 15, HB33T = 38, HB46T = 7, HB70 = 11; exome data was not available for HB66).

### Delimiting Genomic Regions Within Frequent CNAs and Encompassed Genes

**Figure 4:**
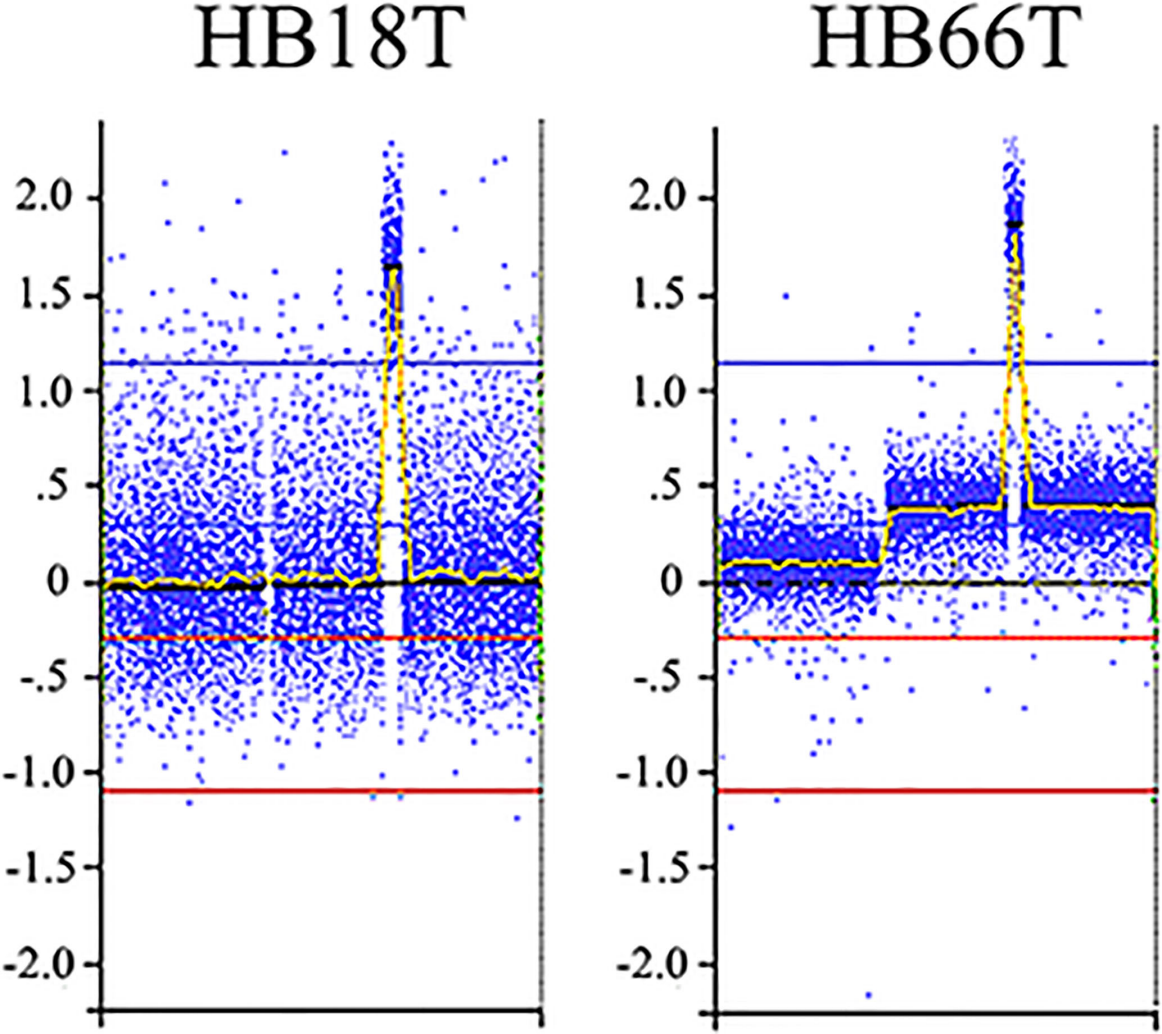
2q24.2q24.3 amplification detected in two HBs. Microarray probes are plotted according to their genomic coordinates of the affected segment on chromosome 2, from the short arm to the long arm. The Y axis represents the log_2_ scale of the copy number ratio tumor/control (values close to 0 indicate regions with similar copy number between tumor and control samples, and positive values represent gains). Note the amplification regions as the highest peaks in the graphs.

We delimited genomic regions of the CNAs most commonly shared by the 16 HBs with detectable alterations. Six genomic regions (25% frequency cut-off; p value < 0.05) were delimited (1p36.33p35.1, 4p14, and 4q21.22q25 losses, and 1q31.3q42.3, 2q23.3q37.3, and 20p13p11.1 gains). **Table 3** presents details of these regions, including genomic coordinates, size, number of encompassed genes, and genes previously reported in COSMIC. In particular, 1p loss was the most frequent deletion, specifically a 34 Mb 1p36.33p35.1 segment shared by ~27% of tumors. Regarding the COSMIC genes encompassed by these regions, four of them were already reported to be somatically mutated in HBs (*PAX7*, *ARID1A*, *H3F3A*, and *NFE2L2*), and 12 presented expression or epigenetic changes in these tumors (*RPL22*, *CAMTA1*, *MDM4*, *ELK4*, *SLC45A3*, *CHN1*, *HOXD13*, *HOXD11*, *CREB1*, *IDH1*, *ACSL3*, and *TET2*). We also identified a minimum common region of an amplification detected in two tumors (**Figure 4**), comprising a 5.3 Mb segment at 2q24.2q24.3 (chr2:161698894-167064256; GRCh37/hg19), presenting at least six copies.

**Table 3:** The six minimum regions delimited within the most frequent CNAs detected in the HB cohort (genomic coordinates given according to GRCh37/hg19).

Cytoband (genomic coordinates)	CNA type	Size (Mb)	Number of genes (COSMIC* genes)
1p36.33p35.1 (1_34142970)	Loss	34	689 (*TNFRSF14, PRDM16, RPL22, CAMTA1, SDHB, PAX7, MDS2, ARID1A, LCK*)
1q31.3q42.3 (197951701_235005704)	Gain	37	407 (*MDM4, ELK4, SLC45A3, H3F3A*)
2q23.3q37.3 (152055371_243199373)/ 2q24.2q24.3 (161698894_167064256)	Gain/Amplification	91/5,3	728 (*CHN1, HOXD13, HOXD11, NFE2L2, PMS1, SF3B1, CREB1, IDH1, ATIC, FEV, PAX3, ACSL3*)/ 33 (0)
4p14 (39452275_40202974)	Loss	0,75	12 (0)
4q21.22q25 (83145880_109498009)	Loss	26	154 (*RAP1GDS1, TET2*)
20p13p11.1 (35547_26376162)	Gain	26	262 (0)

*COSMIC, Catalogue of Somatic Mutations in Cancer Genes - https://cancer.sanger.ac.uk/cosmic.

The six delimited regions encompassed a total of 2,252 genes which were evaluated for their potential contribution to liver tumor biology (see *Materials and Methods*). The 229 genes with the highest scores (top 10% cut-off; score > 6; **Supplementary Table 3**) were submitted to an *in-silico* analysis using GeneAnalytics, with enrichment information regarding Diseases, Pathways, GO Terms, HPO Phenotypes, MGI Phenotypes and others (**Supplementary Table 4**). The top 10 diseases associated with the list of 229 genes were all cancers: hepatocellular, breast, colorectal, lung (and lung susceptibility), prostate, ovarian, gastric, pancreatic, and medulloblastoma (**Supplementary Table 4A**). The most relevant disclosed pathways, presenting with the highest scores (**Supplementary Table 4B** shows the top 50 highest scores), were ERK Signaling, MicroRNAs in Cancer, PI3K-Akt and Akt Signaling, Pathways in Cancer, Mesenchymal Stem Cells and Lineage-specific Markers, PEDF Induced Signaling, Human Embryonic Stem Cell Pluripotency, IL-2, and Proteoglycans in Cancer; these 10 highest scores identified for pathways and respective associated coding and non-coding genes can be found in **Table 4**. The **Supplementary Table 4C** detailed the observed top 50 enriched GO biological processes in HBs with their respective associated genes, while **Supplementary Table 4D** presents data relative to Positive/Negative regulation of GO Biological process. **Table 5** presents the top 15 highest scores and the respective genes associated with the top 15 GO biological process in HBs; the 132 unique genes which are related to these enriched biological processes are separately listed in **Table 6**.

**Table 4:** List of the top 10 enriched pathways of the set of 229 genes used in the *in-silico* analysis.

Pathway	Genes	Score
ERK Signaling	*ACVR1, CASP10, CASP8, IL10, TNFRSF1B, LAMB3, BMP2, COL3A1, CFLAR, PRKCZ, CAPN9, CD28, LEF1, CREB1, STAT1, NRP2, FZD7, STAT4, CDC25B, DES, PSEN2, STMN1, H3-3A, CXCR1, CXCR2, IL24, TP53BP2, ATF2, ERBB4, FZD5, E2F2, ITGA6, CASP9, DVL1, FN1, GNB1, GNRH2, NFKB1, CCL20, EPHB2, TGFB2, HSPG2, PLCB1, CDC42, BMPR2, TNFRSF8, TNFRSF9, ATF3, MAPK10, MTOR, IRS1, ITGAV, SPP1, EPHA2, COL4A3, EPHA4*	69.17
MicroRNAs in Cancer	*MIR215, MIR34A, MIR26B, MIR29C, MIR205, MIR29B2, MIR181B1, HDAC1, MIR375, MIR200A, PIK3CD, CDC25B, STMN1, SLC45A3, HDAC4, E2F2, MIR181A1, MDM4, MIR103A2, MIR200B, WNT3A, MIR10B, NFKB1, TGFB2, MIR135B, BMPR2, MTOR, IRS1*	56.69
PI3K-Akt Signaling Pathway	*CASP8, LAMB3, HDAC1, PRKCZ, IBSP, PIK3CD, WNT4, CREB1, STAT1, FZD7, ATF2, ERBB4, FZD5, JAG1, ITGA6, CASP9, DVL1, WNT10A, FN1, IKBKE, GNB1, WNT3A, NFKB1, WNT9A, WNT6, CDC42, MTOR, IRS1, ITGAV, SPP1, EPHA2, COL4A3, EIF4E*	51.42
Pathways in Cancer	*CASP8, LAMB3, BMP2, HDAC1, LEF1, PIK3CD, WNT4, STAT1, FZD7, STAT4, FZD5, E2F2, JAG1, ITGA6, CASP9, DVL1, WNT10A, FN1, GNB1, WNT3A, NFKB1, WNT9A, WNT6, TGFB2, RASSF5, PLCB1, CDC42, NFE2L2, MAPK10, MTOR, ITGAV, AGT, COL4A3*	51.00
Akt Signaling	*ACVR1, IL10, TNFRSF1B, TLR5, BMP2, PRKCZ, CD28, LCK, CREB1, STAT1, STAT4, CXCR1, CXCR2, IL24, ATF2, ERBB4, ITGA6, CASP9, IKBKE, GNB1, GNRH2, NFKB1, CCL20, EPHB2, TGFB2, PLCB1, CDC42, BMPR2, TNFRSF8, TNFRSF9, MAPK10, MTOR, IRS1, ITGAV, EPHA2, EIF4E, EPHA4*	51.00
Mesenchymal Stem Cells and Lineage-specific Markers	*BMP2, MYOG, ALPP, WNT4, TNNT2, DES, PAX7, PTPRC, PAX3, FN1, CD34, TGFB2, FABP3, HSPG2, SPP1*	42.21
PEDF Induced Signaling	*ACVR1, CASP10, CASP8, IL10, TNFRSF1B, BMP2, HDAC1, CFLAR, PRKCZ, CD28, CREB1, INHA, ARID1A, CXCR1, CXCR2, IL24, TP53BP2, ERBB4, SMARCAL1, CASP9, IKBKE, NFKB1, CCL20, EPHB2, TGFB2, PLCB1, BMPR2, TNFRSF8, TNFRSF9, MAPK10, MTOR, EPHA2, PLA2G2A, EPHA4*	40.06
Human Embryonic Stem Cell Pluripotency	*ACVR1, BMP2, PRKCZ, LEF1, WNT4, FZD7, ATF2, FZD5, E2F2, DVL1, WNT10A, GNB1, WNT3A, WNT9A, NPPA, WNT6, TGFB2, BMPR2*	38.54
IL-2 Pathway	*TLR5, PRKCZ, LCK, CREB1, CR2, STAT1, STAT4, PSEN2, ATF2, ERBB4, PTPRC, E2F2, IKBKE, NFKB1, PLCB1, CDC42, ATF3, MAPK10, MTOR, IRS1, SPP1, MAP3K6*	36.45
Proteoglycans in Cancer	*PIK3CD, WNT4, HPSE, FZD7, ERBB4, FZD5, WNT10A, FN1, WNT3A, MIR10B, WNT9A, WNT6, TGFB2, HSPG2, CDC42, GPC1, MTOR, ITGAV*	36.26

**Table 5:** List of the top 15 enriched GO Biological process of the set of 229 genes used in the *in-silico* analysis.

Biological process	Genes	Score
Positive Regulation of Transcription, DNA-templated	*ACVR1, IL10, ESRRG, PROX1, BMP2, HDAC1, PRDM2, MYOG, LEF1, WNT4, CREB1, PRDM16, STAT1, FZD7, ARID1A, HDAC4, ERBB4, RUNX3, MAD2L2, PAX3, DVL1, WNT3A, NFKB1, WNT6, ELF3, PLCB1, NFE2L2, SPP1, TP73, AGT*	35.55
Negative Regulation of Cell Proliferation	*IL10, MIR29C, PROX1, MIR29B2, BMP2, MYOG, FRZB, IL24, HDAC4, ERBB4, FZD5, PDPN, MDM4, RBBP4, WNT9A, TGFB2, FABP3, RASSF5, PTPN14, PTPRU, TNFRSF8, TNFRSF9, KISS1, COL4A3*	32.23
Positive Regulation of Gene Expression	*MIR34A, MIR29B2, RPL11, BMP2, CD28, NR0B2, LEF1, PIK3CD, AVP, SLC11A1, ATF2, CD46, WNT10A, FN1, WNT3A, CD34, EPHB2, WNT6, TTN, NFE2L2, KDM5B, KIF1B, ATF3, MTOR*	30.15
Canonical Wnt Signaling Pathway	*LEF1, WNT4, FRZB, FZD7, FZD5, DVL1, WNT10A, WNT3A, WNT9A, WNT6*	27.04
Positive Regulation of Cell Proliferation	*PROX1, CST3, HDAC1, PRKCZ, LEF1, AVP, STAT1, CDC25B, CXCR2, IL24, HDAC4, ERBB4, DPP4, CSNK2A1, FN1, MIR200B, WNT3A, TGFB2, CUL3, ATF3, IRS1, ITGAV, AGT*	24.73
Positive Regulation of Transcription By RNA Polymerase II	*ACVR1, IL10, ESRRG, PARP1, PROX1, BMP2, HDAC1, PRDM2, MYOG, CD28, LEF1, CREB1, STAT1, STAT4, SLC11A1, KDM1A, PAX7, HDAC4, ATF2, FZD5, E2F2, SATB2, JAG1, ITGA6, PAX3, CAMTA1, WNT3A, NFKB1, HOXD13, ELF3, BMPR2, TET2, NFE2L2, ATF3, TP73*	24.21
Apoptotic Process	*CASP10, CASP8, TNFRSF1B, PARP1, MFN2, CST3, FAP, CFLAR, AVP, IL24, TP53BP2, ERBB4, PDCD1, CDCA7, CASP9, PAX3, SNCA, CSNK2A1, NFKB1, RASSF5, CHI3L1, TNFRSF9, KIF1B, TP73, EPHA2*	22.98
Negative Regulation of Gene Expression	*MIR29C, MIR29B2, BMP2, HDAC1, CD28, NR0B2, PIK3CD, WNT4, CREB1, TARDBP, MIR181A1, CD46, MIR200B, CD34, NFKB1, TGFB2*	21.99
Viral Process	*CASP8, CFLAR, LCK, CREB1, CR2, STAT1, XRCC5, NRP2, PCNA, PTPRC, CR1, CD46, IKBKE, DYNC1I2, HSPD1, NFE2L2, SPEN, ITGAV, TP73, EPHA2, EIF4E, WASF2*	21.82
Response to Drug	*PMS1, IL10, CST3, LCK, CREB1, STAT1, XRCC5, IGFBP2, HDAC4, MTHFR, REN, SNCA, UGT1A1, TGFB2, FABP3, TP73*	21.68
Atrioventricular Valve Morphogenesis	*ACVR1, BMP2, MDM4, TGFB2, BMPR2*	21.48
Response to Lipopolysaccharide	*CASP8, IL10, TNFRSF1B, ALPL, SLC11A1, REN, CASP9, SNCA, UGT1A1, THBD, HSPD1, TFPI*	21.10
Negative Regulation of Transcription By RNA Polymerase II	*PER2, PARP1, PROX1, BMP2, HDAC1, PRDM2, NR0B2, LEF1, PRDM16*, *STAT1, ARID1A, ENO1, PCNA, KDM1A, HDAC4, ATF2, E2F2, SATB2*, *RUNX3, MAD2L2, MDM4, SNCA, NFKB1, SPEN, CUL3, ATF3, MAPK10*	20.64
Positive Regulation of Apoptotic Process	*CTLA4, MAP3K20, MIR29C, MIR29B2, BMP2, CREB1, FRZB, PSEN2, PDCD1, ITGA6, CASP9, SNCA, MIR200B, HSPD1, TNFRSF8, TP73, BARD1*	20.23
Wnt Signaling Pathway	*LEF1, WNT4, FRZB, FZD7, FZD5, CSNK2A1, DVL1, WNT10A, WNT3A, WNT9A, WNT6, CUL3, LGR6*	19.45

**Table 6:** List of the 132 unique genes which are related to the top 15 enriched biological processes detected in hepatoblastomas.

*ACVR1*	*E2F2*	*MAD2L2*	*PTPRC*
*AGT*	*EIF4E*	*MAP3K20*	*PTPRU*
*ALPL*	*ELF3*	*MAPK10*	*RASSF5*
*ARID1A*	*ENO1*	*MDM4*	*RBBP4*
*ATF2*	*EPHA2*	*MFN2*	*REN*
*ATF3*	*EPHB2*	*MIR181A1*	*RPL11*
*AVP*	*ERBB4*	*MIR200B*	*RUNX3*
*BARD1*	*ESRRG*	*MIR29B2*	*SATB2*
*BMP2*	*FABP3*	*MIR29C*	*SLC11A1*
*BMPR2*	*FAP*	*MIR34A*	*SNCA*
*CAMTA1*	*FN1*	*MTHFR*	*SPEN*
*CASP10*	*FRZB*	*MTOR*	*SPP1*
*CASP8*	*FZD5*	*MYOG*	*STAT1*
*CASP9*	*FZD7*	*NFE2L2*	*STAT4*
*CD28*	*HDAC1*	*NFKB1*	*TARDBP*
*CD34*	*HDAC4*	*NR0B2*	*TET2*
*CD46*	*HOXD13*	*NRP2*	*TFPI*
*CDC25B*	*HSPD1*	*PARP1*	*TGFB2*
*CDCA7*	*IGFBP2*	*PAX3*	*THBD*
*CFLAR*	*IKBKE*	*PAX7*	*TNFRSF1B*
*CHI3L1*	*IL10*	*PCNA*	*TNFRSF8*
*COL4A3*	*IL24*	*PDCD1*	*TNFRSF9*
*CR1*	*IRS1*	*PDPN*	*TP53BP2*
*CR2*	*ITGA6*	*PER2*	*TP73*
*CREB1*	*ITGAV*	*PIK3CD*	*TTN*
*CSNK2A1*	*JAG1*	*PLCB1*	*UGT1A1*
*CST3*	*KDM1A*	*PMS1*	*WASF2*
*CTLA4*	*KDM5B*	*PRDM16*	*WNT10A*
*CUL3*	*KIF1B*	*PRDM2*	*WNT3A*
*CXCR2*	*KISS1*	*PRKCZ*	*WNT4*
*DPP4*	*LCK*	*PROX1*	*WNT6*
*DVL1*	*LEF1*	*PSEN2*	*WNT9A*
*DYNC1I2*	*LGR6*	*PTPN14*	*XRCC5*

Moreover, the analyzed set of 229 genes was found to be relevant for several neoplasms (liver, gastrointestinal and genitourinary tracts, hematological, skin and vascular) as well as abnormality of the liver function and morphology (**Supplementary Table 4E**). The analysis considering the association of these genes with reported mouse phenotypes highlighted abnormal embryonic development, embryonic lethality during organogenesis and premature death, decreased cell proliferation, decreased body weight and embryo size, and abnormal cytokine secretion (**Supplementary Table 4F**).

### Literature Review

We compiled data from 45 studies, which investigated the entire genome of 480 primary HBs; samples with no detectable alterations (n = 141) were excluded. This literature review was based on CNA data obtained with techniques with different resolution (karyotype, FISH, CGH, and genomic microarrays; **Supplementary Table 5**), and CNAs were grouped only according to chromosome arm level; the results from Rodrigues et al. (12) were not included, since the analysis of the respective tumors was expanded at higher resolution and presented together with the entire Brazilian cohort, reported here.

**Figure 5:**
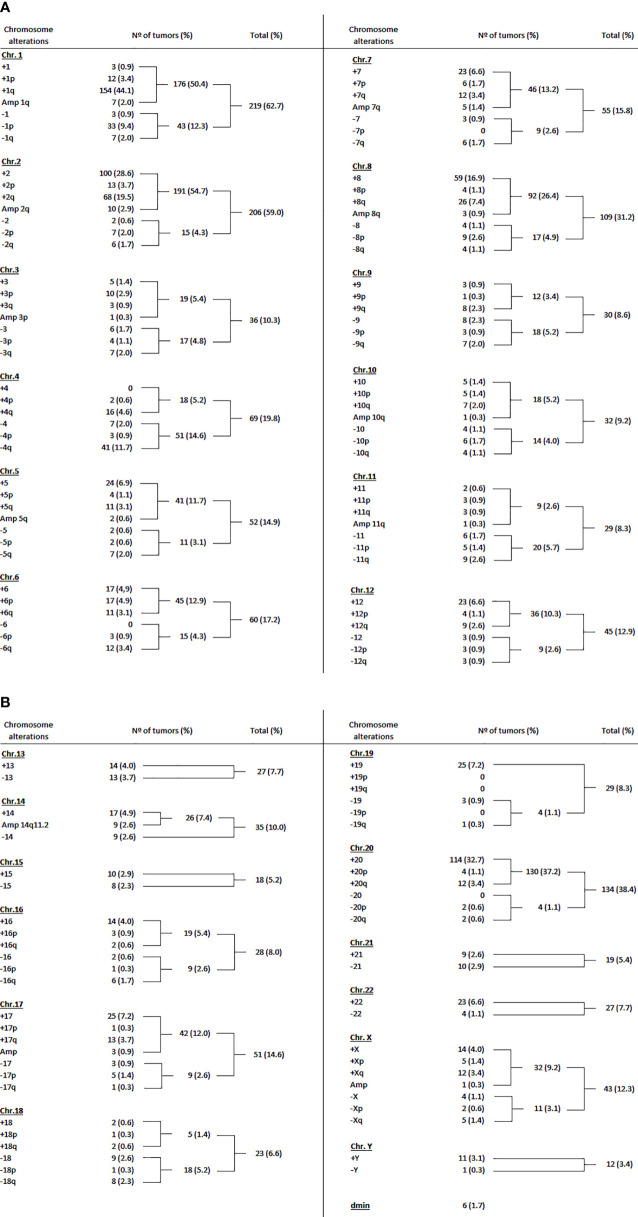
Literature review of the cytogenomic data on hepatoblastomas. **(A)** Summary of the reported hepatoblastoma copy number chromosomal alterations in the literature from 1985 to 2017: chromosomes 1 to 12. **(B)** Summary of the reported hepatoblastoma copy number chromosomal alterations in the literature from 1985 to 2017: chromosomes 13 to 22, X, Y, and dmin. All references were these data were based can be found in the **Supplementary Table 5**.

The chromosomes most frequently reported to be altered in the 349 HBs with cytogenetic findings according to our review were chromosomes 1 (50.4%), 2 (54.7%), 8 (26.4%) and 20 (37.2%) for gains, while losses were predominant for chromosomes 1 (12.3%) and 4 (14.6%) (**Figures 5A, B**). The most recurrent alterations were typically restricted to specific chromosome arms, such as 1q (46.1%), 2q (22.4%) and 8q (8.3%) for gains, while the most frequent losses were located at 1p (9.4%) and 4q (11.7%). Moreover, gains affecting the chromosomes 5, 6, 7, 12 and 17 were present in this analysis in a frequency ranging from 10-13%.

**Figure 6:**
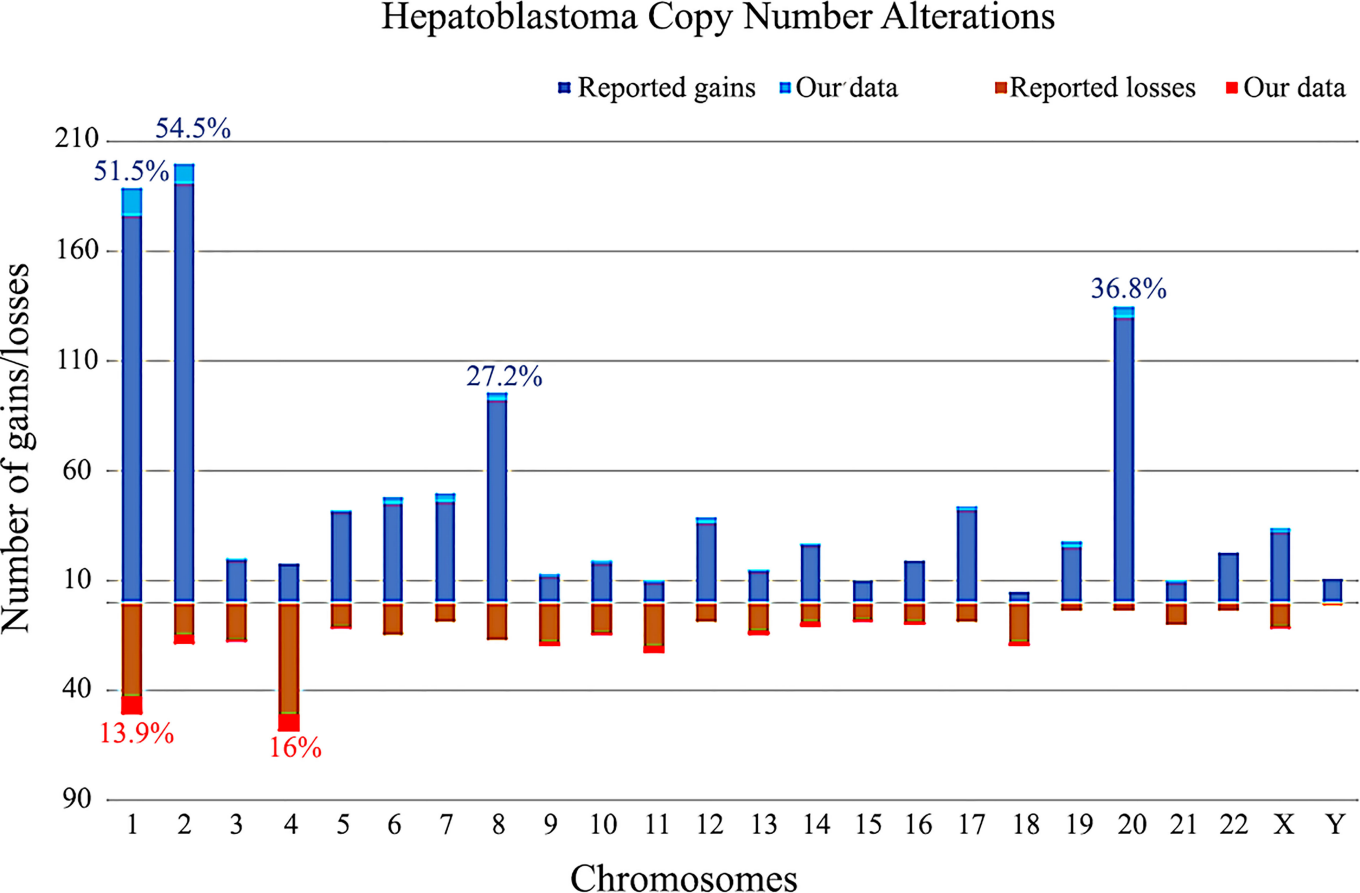
Copy number gains and losses distributed per chromosome in 365 hepatoblastomas, based on our review of literature and the present study. Columns above the X axis represent the total number of gains per chromosome; below the X axis, the total number of losses. The frequencies of the most commonly chromosomal alterations detected in HBs in the literature along with our data are presented as percentages.

The CNA data on 349 HBs from the literature and 16 samples from the present study are summarized in **Figure 6**. Taken together, the most recurrent CNAs in these 365 HBs were gains at 1 (51.5%), 2 (54.5%), 8 (27.2%) and 20 (36.8%), and chromosomes 1 (13.9%) and 4 (16%) losses.

## Discussion

The analysis of chromosome alterations provides clues regarding biological pathways related to specific cancer types, and can have implications in tumor diagnosis and risk stratification, besides being used as prognostic markers in some cases (16). Therefore, the study of cancer genomes can help not only to understand the general biology, but also to differentiate subgroups within the same type of cancer. The major aim of this study was to identify CNA hot-spot regions in HB, possibly linked to clinical data, and at the same time compile the cytogenetic available literature data on this tumor, due to a lack of information on this tumor type in all cancer databases.

At the best of our knowledge, about 50 studies have been published reporting numerical and structural chromosomal alterations in HB tumors, comprising ~500 tumors that were characterized over a period of approximately 30 years. Among these studies, 45 investigated the entire genome of 480 primary HB tumors. In the present literature review, studies involving cell lines were excluded, but it is worth mentioning the papers from Doi (17) and Miyamoto et al. (18), who established and cytogenetically characterized, respectively, the hepatoblastoma cell line HUH-6-clone 5, with modal karyotype: 49, XY, +i(8q), +12, +20, producing the first cytogenetic information described in the literature on this type of tumor. It is important to point out that this is a compilation of data obtained with different techniques; therefore, this analysis precludes the precise mapping of CNAs based on cytobands. For this reason, CNAs were grouped only according to the chromosome arm where the alteration was located. Besides, HB genomes mostly carry aneuploidies and chromosome arm alterations, with a limited number of small CNAs. Also, tumors with no detectable alterations (n = 141, including ours) were removed from this review.

Literature data have been reporting that the most common CNAs in HBs were gains of 1q, 2/2q, 8/8q and 20; and 4q and 18 losses. Our review confirmed the literature reports for the most prevalent gains; however, the chromosome 18 loss did not stand out as a frequent event. It was noteworthy that 1p was found to be the second most common deletion in HBs in our review; likewise, in the Brazilian cohort, the 1p loss was the most frequent deletion, specifically a 34 Mb 1p36.33p35.1 segment shared by ~27% of the tumors. Therefore, there is a discrepancy regarding the frequency of chromosome 18 loss in HBs between our data and literature review when compared with previous reports. This discrepancy can be attributed to the fact that recent studies on the CNA profile of HBs did not take into account a large number of tumors such as we did (total of 365 HBs with cytogenetic findings). In fact, the last review performed on cytogenetic data was published in 2012 (19), with a smaller number of HBs. Furthermore, the chromosome 18 loss can be linked to a specific cohort, a group of 111 HBs analyzed by (20), in which they found chromosome 18 losses in several samples; probably, the notion that 18 loss is a common alteration in HB was derived from this study and the subsequent revision published in 2012 (19). However, interestingly, we observed in the Brazilian group an enrichment of chromosome 18 loss in high-risk HBs. Sumazin et al. (21) also reported 1p deletion as a frequent alteration in HBs. Deletions in the short arm of chromosome 1, particularly the terminal region, are frequently observed in many cancers, including hepatocellular carcinoma (22), neuroblastoma (23) and rhabdomyosarcoma (24). The frequency of this chromosomal alteration suggests that it is a nonrandom event, probably harboring tumor suppressors relevant for tumorigenesis in different tissues. At least five genes located at 1p36 (*CHD5*, *CAMTA1*, *KIF1B*, *CASZ1*, and *MIR34A*) were already suggested to be tumor suppressors (25).

It is also worth mentioning a recent published study proposing a DNA methylation-based classification for HBs (26), in a cohort of 59 samples; CNAs not commonly found in previous studies were reported, such as gains mapped to chromosomes 5, 6, 7, 12 and 17. Another recent study (6) also reported gains on chromosomes 5, 6, 7, 12 and 17 as frequent alterations in their 34 HBs cohort. These gains are represented along our literature review, in a frequency ranging from 10-13%. Another relevant aspect is that CNA gains, particularly of whole-chromosomes and whole-chromosome arms, occur more often than losses in the published studies. This prevalence of gains was not observed in our HB cohort; however, we did detect a significantly larger median length of the gain events (74.7 Mb) in comparison to losses (2 Mb), pointing to a pattern of prevalence of whole-chromosome/chromosome arms for gains, while the majority of losses were focal alterations.

Gene amplifications are frequently observed in cancer, and are one of the major causes of tumorigenesis driven by gene expression alterations as the result of copy number changes. Amplified regions are known to contain oncogenes and have the potential to serve as prognostic factors and therapeutic targets (27). Two tumors in our cohort, one of them previously reported (12), harbored 2q24 amplifications, a cytoband already described as amplified in other studies (28–32). This amplification has been associated with tumor progression (9, 30), and as indicative of poor prognosis (31). Clinically, the two Brazilian patients whose HBs harbor 2q24 amplifications were males diagnosed under one year of age, with high levels of AFP (> 200,000 ng/mL), and one of them classified as high risk, according to CHIC parameters (13, 14). We could restrict the minimum amplified region to a 5.3 Mb segment at 2q24.2q24.3, harboring 33 genes; however, the smallest 2q24 amplified region identified in HBs was documented by Kato et al. (30) a ~2 Mb region (chr2: 162954232-164891865; hg19), fully contained in our delimited region, and encompassing only eight genes (*LOC101929532*, *GCG*, *FAP*, *IFIH1*, *GCA*, *KCNH7*, *KCNH7-AS1*, *FIGN*). Two genes mapped on the larger 5.3 Mb amplification region could be highlighted: *FAP*, expressed mainly during embryogenesis, and in cancer-associated fibroblasts (33), and *DPP4*, which is overexpressed in several tumors and described as having a pro-oncogenic role in hepatocellular carcinoma (34). We have previously profiled the mRNA expression of 48 genes mapped to a 10 Mb 2q24 amplification (12), a larger segment encompassing ours and the smallest 2q24 amplified region documented by Kato et al. (30); only five genes were found to be significantly upregulated in the investigated tumors (*DAPL1, ERMN, GALNT5, SCN1A* and *SCN3A*).

In general, in the Brazilian group, HBs from categories such as females, high risk tumors, tumors who developed in older patients or from patients with metastasis and/or deceased, carried a higher diversity of chromosomal alterations scattered along the genome and not restricted to the reported recurrent regions. In particular, these tumors exhibited mainly chromosomal losses at 1p, 4, 11q and 18q, indicating that losses at these regions can be of worst prognosis.

According to the identified CNA profiles, we distinguished three major groups in our casuistry: no detectable CNA, few CNAs and tumors with complex genomes. Due to the small number of samples, only few significant clinical associations were disclosed in relation to these three CNA profiles. Still, some clinical differences can be pointed out. Tumors with simpler genomes (mainly no detected CNAs) exhibited a significant association with the epithelial fetal subtype of HB. Moreover, HBs with low CNA complexity were mainly stratified as low/intermediate risk (65% of the samples), and mostly observed in patients without relapse and metastasis.

In contrast, the complex genome group included three cases with epithelial embryonal histology, as well as the only HB with HCC features. Of note, we detected a significant association of complex genomes with older patients who developed high-risk tumors, with metastasis and poor outcome. The highest number of CNAs were detected in the HB46 and HB70 samples (27 and 24 CNAs, respectively); however, the reason for this discrepancy even compared to the group of complex genomes is not clear. Similarly, two patients with HB exhibiting complex genomes were clinically unusual; one was the only case of a congenital HB, who also was born with unilateral renal agenesis, while other presented global developmental delay and complex craniosynostosis. The association between congenital anomalies and pediatric cancer is well-documented in the literature (35), and another two HB patients of this cohort were born with congenital kidney abnormalities. Among these four HB patients with congenital anomalies, two of them exhibited complex genomes.

Together, these findings suggest that chromosomal losses, particularly 1p and 18 losses, and a high load of CNAs, increase the tendency to HB aggressiveness, and the presence of the sample with HB/HCC features in this group is in agreement with this hypothesis (36). To further explore this possibility, we integrated somatic CNA and SNV/indel data of overlapping tumors (15). We could observe that HBs which exhibited complex genomes also had a higher number of somatic coding mutations in comparison to the whole group; indeed, HBs with no chromosomal alterations presented with the lowest number of somatic mutations in our exome study, suggesting that other mechanisms must be involved in their tumorigenesis, such as epigenetic changes, as we previously hypothesized (37–40), and was corroborated by large recent studies (6, 26, 41). In addition, different metabolic profiles were shown to be HB biomarkers associated with *CTNNB1* mutations and histological subtypes, disclosing an underlying pathway with relevant clinical implications (42).

Six chromosome regions were delimited in our cohort (1p36.33p35.1, 1q31.3q42.3, 2q23.3q37.3, 4p14, 4q21.22q25 and 20p13p11.1), and the encompassed genes were evaluated for their potential contribution to tumor biology, using *in-silico* analytic tools. Interestingly, the enrichment analysis showed that the most enriched biological pathways were the ERK Signaling, MicroRNAs in Cancer, and the PI3K/AKT Signaling, in addition to the well-known WNT Signaling pathway, listed several times in the analysis, but surprisingly not presenting the highest scores.

Disruptions in the ERK pathway have been associated with many developmental abnormalities as well as with cancer predisposition (43). Mutations resulting in the hyperactivation of ERK are frequently observed in many types of cancer, and the translocation of ERK into the cell nucleus seems to play an important role in the oncogenic process and cell proliferation (44). The PI3K/AKT pathway is also frequently mutated in many cancer types, and it is responsible for several cellular functions related to oncogenesis (45). In a previous work, we also found enrichment of the MAPK and WNT signaling pathways in HBs, suggesting modulation by methylation changes (37). Although studies (46–49) have reported dysregulations related to ERK and PI3K/AKT pathways in HB, further investigation is needed to detail how these pathways are contributing to HB tumorigenesis, possibly not directly linked to WNT. A previous work from our group (15) performed whole-exome sequencing in some samples herein reported, and a *ERBB4* mutation, a gene related to ERK and AKT pathways, was detected. Additionally, the enriched biological pathways observed in this work also reinforce the occurrence of deregulation of the chemokine family in HB, as we previously reported (15). The association of chemokines with liver cancer has been increasingly studied, because most of them are involved in activation of the immunological cascades in tumors, frequently linked to worse prognosis and metastasis (50–53).

Based on the identified pathways in this study, we checked for genes or pathways that are drug targets in children and adolescents participating in the ongoing MATCH clinical trial (NCI-COG Pediatric MATCH - National Cancer Institute (https://www.cancer.gov/about-cancer/treatment/clinical-trials/nci-supported/pediatric-match, accessed on 2021-09-24) (54). Indeed, we found three drug targets currently under trial in MATCH: *IDH1* (inhibitor ivosidenib), P13K/mTOR (LY3023414), and PARP (olaparib). Moreover, a talk between the PI3K/AKT and MAPK/ERK pathways has been reported and is probably related to cell survival regulation response; co-targeting and inhibition of these pathways has been shown effective in specific cancer treatments (55), and could be a possible new strategy for HB. Nevertheless, the relevance of these drugs for HB treatment is yet unknown.

Our data also highlighted an enrichment in the biological pathway MicroRNAs in Cancer, since several miRNA genes were mapped in the most frequent alterations in this HB cohort, in particular a cluster at 1q32. miRNAs can act as oncogenes or tumor suppressors and may have opposite functions depending on the cancer type (56). Studies exploring miRNAs in HB are scarce, but a recent review (57) pointed to some miRNAs which participate in key signaling pathways, such as WNT, Myc and Hippo.

Carrying out studies with HBs is challenging, mainly due to its rarity, thus resulting in lack of information about this tumor in cancer databases. In this work, we reviewed the data on HB cytogenetics and cytogenomics available in the literature, and also described the genomic CNA profile of a Brazilian cohort of 26 HBs. Even with the resolution limitations inherent to the techniques used in these studies, the most prevalent CNAs in HB were described, and new chromosome segments of interest were highlighted. Our analysis revealed diverse biological pathways that could be related to HB tumorigenesis, although certainly waiting for validations.

## Data Availability Statement

The original contributions presented in the study are included in the article/**Supplementary Material**. Further inquiries can be directed to the corresponding author.

## Ethics Statement

The studies involving human participants were reviewed and approved by Hospital das Clínicas/FMUSP - CAAE 47277115.0.0000.0068, and A. C. Camargo Cancer Center Research Ethics Committee - number 1987/14. Written informed consent to participate in this study was provided by the participants’ legal guardian/next of kin. Written informed consent was obtained from the minor(s)’ legal guardian/next of kin for the publication of any potentially identifiable images or data included in this article.

## Author Contributions

Conceptualization: AK. Methodology JB, SC, TA,MR, MC, SRCT, EMN, VO, LMC, DMC, IWC, CMLC, and AK. Validation, JB, TA, AK. Formal analysis, JB,TA. Investigation, JB, TA, AK. Resources, JB, MC, ST, EN, VO, LC, DC, IC, CC, CR, and AK. Data curation, JB, SC, TA,MR, MC, SRCT, EMN, VO, LMC, DMC, IWC, CMLC, and AK. Writing—original draft preparation, JB. Writing—review and editing, JB, TA, CR, AMVM, AK Supervision, AK. Funding acquisition, CR, AMVM, AK. All authors contributed to the article and approved the submitted version.

## Funding

This research was carried out with financial support from CAPES (Brazilian Federal Agency for Support and Evaluation of Graduate Education) project number 2017/1671499; and FAPESP (São Paulo Research Foundation) project numbers: 2019/17423-8, 2018/22893-0, 2018/21047-9 and CEPID - Human Genome and Stem-Cell Research Center - 2013/08028-1.

## Conflict of Interest

The authors declare that the research was conducted in the absence of any commercial or financial relationships that could be construed as a potential conflict of interest.

## Publisher’s Note

All claims expressed in this article are solely those of the authors and do not necessarily represent those of their affiliated organizations, or those of the publisher, the editors and the reviewers. Any product that may be evaluated in this article, or claim that may be made by its manufacturer, is not guaranteed or endorsed by the publisher.

## Supplementary Material

Supplementary Figure 1 CNAs profile comparison between the tumors with *CTNNB1* activation and negative tumors. No remarkable differences were seen. The X axis displays chromosome mapping from 1 to 22 and X, chromosome Y was not evaluated. Copy number gains are represented in blue bars and losses in red; the width of the bars indicates the frequency of the alteration in the group.
