# Supplementary figures and images for: Copy Number Alterations in Hepatoblastoma: Literature Review and a Brazilian Cohort Analysis Highlight New Biological Pathways
Source: Front Oncol. 2021 Dec 8;11:741526. doi: 10.3389/fonc.2021.741526 (PMC8692715; doi:10.3389/fonc.2021.741526)

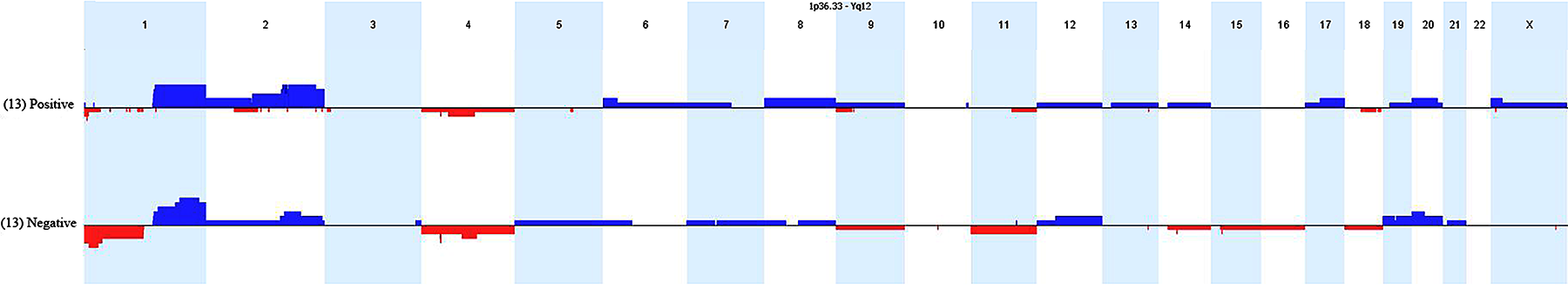

Supplement: Supplementary Figure 1 — CNAs profile comparison between the tumors with CTNNB1 activation and negative tumors. No remarkable differences were seen. The X axis displays chromosome mapping from 1 to 22 and X, chromosome Y was not evaluated. Copy number gains are represented in blue bars and losses in red; the width of the bars indicates the frequency of the alteration in the group. [file Image_1.tif]
